# Supplementary material for: Phylogenetic Analysis of Newcastle Disease Virus Isolated from Poultry in Live Bird Markets and Wild Waterfowl in Zambia
Source: Microorganisms. 2024 Feb 8;12(2):354. doi: 10.3390/microorganisms12020354 (PMC10893471; doi:10.3390/microorganisms12020354)
Supplement: Supplementary file 1 [file microorganisms-12-00354-s001.zip › microorganisms-2843749-supplementary.pdf]

Supplementary Materials

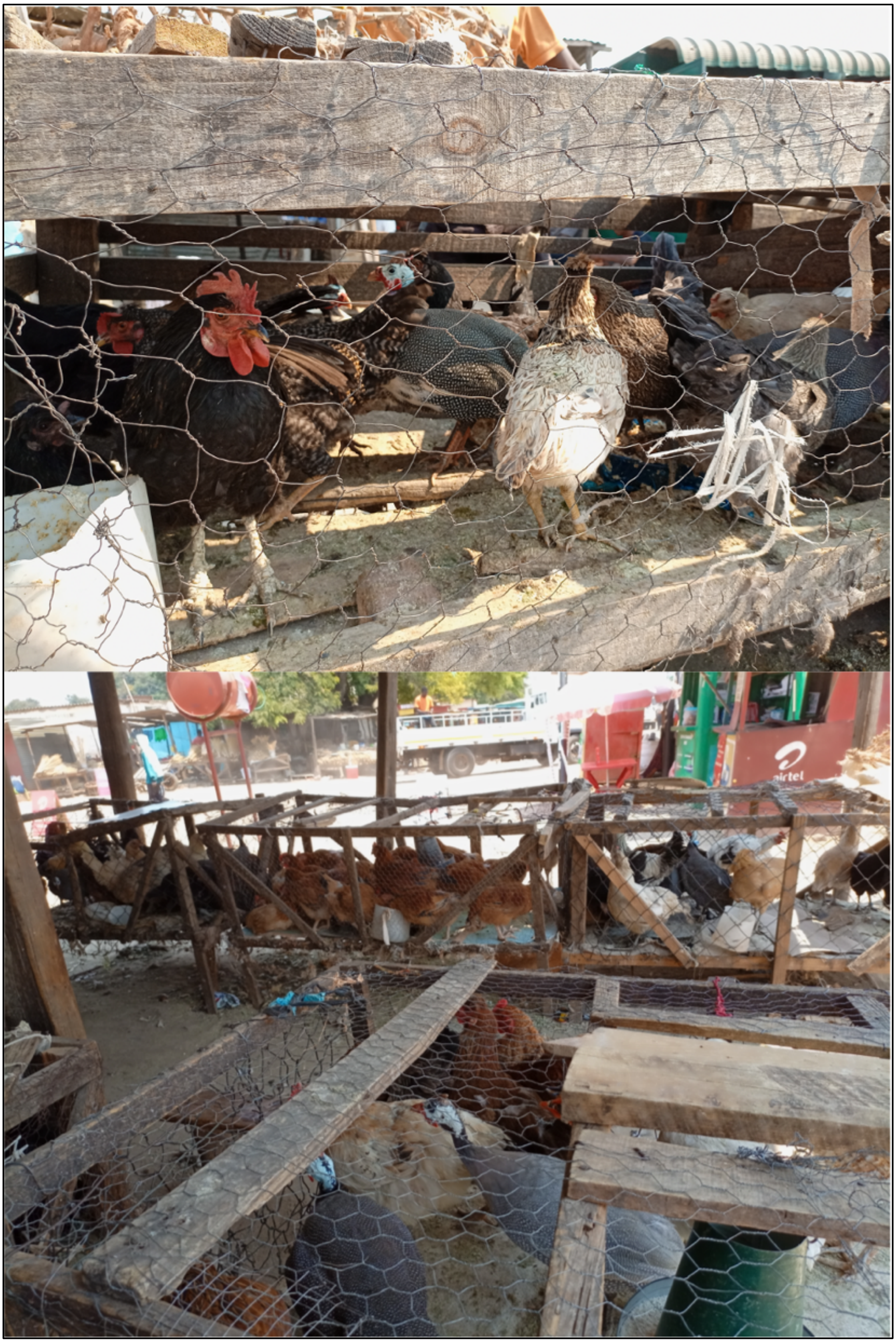

Figure S1: Mixed poultry in wire mesh cages at one of the live bird markets.

Table S1: Ten top hits with the highest sequence identity similarity to the current study

GenBank Accession Numbers	Strain Name	% Similarity	Country
**Poultry Strains**			
MF622042	chicken/Zambia/Mbeweka/2015	98.4	Zambia
KU523528	NDV/chicken/Mozambique/658/2012	97.7	Mozambique
KX231366	NDV/chicken/Mozambique/1205/2011	97.6	Mozambique
MF622035	chicken/South Africa/230665/2013	97.5	South Africa
MF622045	chicken/South Africa/RBNW-1/2013	97.4	South Africa
KU523526	NDV/chicken/Mozambique/491/2012	97.4	Mozambique
MF622040	chicken/South Africa/Inchanga/2013	97.4	South Africa
MF622037	chicken/South Africa/239391/2013	97.4	South Africa
KR074404	IBS002/11	97.4	Malaysia
KR815908	turkey/South Africa/N2057/2013	97.4	South Africa
**Strain 708/2021**			
MW927496	mallard/Buryatia/Russia/96i/2019	98.9	Russia
MW927501	gadwall/Amur region/Russia/6b/2019	98.7	Russia
MW927497	common teal/Buryatia/Russia/28i/2019	98.4	Russia
MW927492	common teal/Primorje/Russia/188/2019	98.4	Russia
MZ825554	Northern pintail/Novosibirsk region/Russia/513k/2018	98.4	Russia
MZ666231	slaty-backed gull/Kamchatka/Russia/111/2013	98.4	Russia
MZ802814	Anseriformes/Taiwan/AHRI177/2020	98.4	Taiwan
KC503412	Anas sp/Japan/10UO0343/2010	98.4	Japan
MZ666228	shoveler/Kazakhstan/30kz/2014	98.3	Kazakhstan
MZ802812	Anseriformes/Taiwan/AHRI158/2019	98.3	Taiwan
**Strain 1636/2015**			
MZ666228	shoveler/Kazakhstan/30kz/2014	98.9	Kazakhstan
MW927501	gadwall/Amur region/Russia/6b/2019	98.9	Russia
MZ666231	slaty-backed gull/Kamchatka/Russia/111/2013	98.9	Russia
AB871656	duck/Tottori/453/2009	98.7	Japan
KC503412	Anas sp/Japan/10UO0343/2010	98.7	Japan
AB871657	duck/Tottori/481/2009	98.7	Japan
MN632509	Anseriformes/Taiwan/AHRI76/2013	98.7	Taiwan
MZ825554	northern pintail/Novosibirsk region/Russia/513k/2018	98.6	Russia
MW927496	mallard/Buryatia/Russia/96i/2019	98.5	Russia
EU493454	Pochard/Finland/13193/06	98.4	Finland
